# Circulation of the Blood—New Motive Power

**Published:** 1854-06

**Authors:** 


### *The Circulation of the Blood—New Motive Power.*

In our exchanges from the North, and also from the South, we notice that considerable inquiry has been excited in regard to the circulation of the blood. The subject also lately came up in the Drake Society of this City, and occupied some three or four evenings in its discussion.

A knowledge of Harvey's discovery seems, at this day of progress in physiological matters, to be nothing more than the mere alpha of the process. The normal amount of blood, its chemical composition, the capacity of the central organ, and the vessels connected with it, to contain and propel the fluid to the various parts of the organism; and the probability that other forces, more potent and better adapted to the purpose, are concerned in the process, are questions that are now exciting attention as being of especial interest to the physiologist, pathologist, and practising physician.

The principal effort of those who have brought the matter forward for consideration at the present time, seems to be, to show that there are other forces, besides that of the heart, to which the movements of the blood are due. The sum of these, Dr. Cartwright proposes to express by the term, Hœmatokinety. To the introduction of this term into physiological science, a formal protest has been entered in certain quarters. Among others who reject it, and mostly for whose benefit it has been supposed it was coined, is Mrs. Willard of Troy, New York, who has made herself quite notorious as the alleged discoverer of what she regards a "*New Motive Power.*"

She insists on the truth of the following doctrines, which we have thrown into form as a kind of substatum for her theory.

1st. That in the process of respiration, combustion takes place from the presence of the oxygen of inhaled air, and the carbon of venous blood, and caloric, as a consequence, is set free. 2d. About a fifth part of all the blood in the system is in the lungs, and about seven-eigths of this is water. 3d. The temperature of the blood in the lungs is about thirty degrees higher than is necessary to convert water *in vacuo* into vapor, and the blood in the lungs is mostly *in vacuo*; 4th. Such being the case, a portion of water in the blood is there changed to vapor, and the volume of the blood becomes so expanded, that it must move. From the influence of the valvular system, the course is directed to the left side of the heart. Possessed of great irritability, this organ as soon as touched by a warm fluid beats, and by its valvular arrangement, the current is propelled forwards.

Dr. Cartwright, who is supposed to have exhibited some sympathy for this theory, has been held up, by several writers of some ability, and formally ridiculed. This has awakened in the Doctor, a disposition to defend himself, and as a consequence, he reviews the whole subject connected with the motive powers of the circulation, and makes some experiments, an account of which is published in the New Orleans Medical and Surgical Journal, that are among the most striking on record.

It is perhaps generally known that Prof. Draper, in a treatise on the forces, which produce the organization of plants, puts forth a physical principle, which is appealed to, by those who are incredulous in regard to the agency of the heart, in capillary circulation. It seems it is susceptible of demonstration, that "if two fluids communicate with one another in a capillary tube, or in a porous or paren-chymatous structure, and have for that tube different chemical affinities, movement will ensue; that liquid which has the most energetic affinity will move with the greatest velocity, and may even drive the other liquid before it." Now then, that arterial blood, charged with oxygen with which it is ready to part, and being prepared to receive in exchange carbonic acid, which the tissues set free—must obviously have a greater affinity for those tissues than venous blood, in which both these changes have already been affected. (Carpenter.) The blood, upon mere physical principles, which enters the systemic capillaries on one side of the net work, must as a consequence drive before it, and expel on the other side the blood which has become venous, while traversing it. Of course, in the pulmonary capillaries opposite affinities would prevail.

Such is the principle, and such the explanation of the circulation by capillary attraction and chemical affinity.

In support of this theory, much has been brought forward in regard to the circulation of sap in vegetables, and the circulation of the blood in inferior animals, in neither of which, is there any central propelling organ. Take as a sample the experiments of Hales: "In experiment XXI, I exposed one of the chief roots of a pear tree in full growth, at a depth of two and a half feet, cut off the point of it, and connected the part of the root left in connection with the stem with a tube, which was filled with water, and closed it with mercury. In consequence of the evaporation from the surface of the tree, the root absorbed the water in the tube with such force, that in six minutes the mercury rose eight inches in the tube. This is equal to a column of water nine feet high."

In some of the lower classes of animals, there appears to be an absence of any central propelling organ. This is true of all of two divisions of the animal kingdom—the Animalia Radiata, and Animalia Articulata. Their organs of respiration are almost universally seated on the surface of the body, and they possess no organ similar in function to that of a heart. The same thing, is in part trne, of several classes of Moluscous and vertebrated animals. In these, although there is a central organ, it seems not to be constructed for propelling purposes.

With reference to all these facts and principles, it may be stated, that they go to make it very plausible, that there are other forces besides those of the heart, to which we might look for an explanation of the chief motive power of the circulation, provided we had no heart. But inasmuch as nearly all vertebrated animals possess this organ, and inasmuch as it is highly endowed with contractile powers, which, according to the experiments of Hales, is adequate to sustain a column of water 7½ feet high, the weight of which would be 4 lbs. 6 oz.; and inasmuch as it appears to be beating away all the time in the chest, in a series of well defined rythmical contractions, that with many, are the *sine qua non* of vitality, it might not be a very unwarrantable physiolofiical conclusion to assign it some little place in the list of motive powers.

That however, the heart with all its power, its *vis a tergo* and its *vis efronte* is adequate to explain everything connected with capillary circulation; the circulation in erectile tissues, the portal circulation, the circulation which presents itself within the cranium, &c., &c., is a position to which no one can yield his assent, when all the facts are carefully examined.— *Ohio Med. & Surg. Journal.*

